# Beyond symptomatic alignment: evaluating the integration of causal mechanisms in matching animal models with human pathotypes in osteoarthritis research

**DOI:** 10.1186/s13075-025-03561-4

**Published:** 2025-05-17

**Authors:** Eva Reihs, Anita Fischer, Iris Gerner, Reinhard Windhager, Stefan Toegel, Frank Zaucke, Mario Rothbauer, Florien Jenner

**Affiliations:** 1 Karl Chiari Lab for Orthopaedic Biology, Department of Orthopedics and Trauma Surgery, Medical University of Vienna, Währinger Gürtel 18 - 20, Vienna, 1090 Austria; 2 Faculty of Technical Chemistry, Technische Universität Wien, Vienna, Getreidemarkt 9/163, 1060 Austria; 3 Ludwig Boltzmann Institute for Arthritis and Rehabilitation, Vienna, Austria; 4 Veterinary Tissue Engineering and Regenerative Medicine Vienna (VETERM), Equine Surgery Unit, University of Veterinary Medicine Vienna, Vienna, Veterinärplatz 1, 1210 Austria; 5 Division of Orthopedics, Department of Orthopedics and Trauma Surgery, Medical University of Vienna, Vienna, 1090 Austria; 6 Dr. Rolf M. Schwiete Research Unit for Osteoarthritis, Orthopaedic University Hospital Friedrichsheim GmbH, Maienburgstr. 2, Frankfurt/Main, 60528 Germany; 7 Ludwig Boltzmann Institute for Arthritis and Rehabilitation, Vienna, Austria

**Keywords:** Osteoarthritis, In vivo models, Stratification, Pathophenotypes, Matchmaking

## Abstract

Osteoarthritis (OA) is a highly prevalent and disabling condition lacking curative treatments, with only symptomatic relief available. Recognizing OA as a heterogenous disorder with diverse aetiologies and molecular foundations underscores the need to classify patients by both phenotypes and molecular pathomechanisms (endotypes). Such stratification could enable the development of targeted therapies to surmount existing treatment barriers. From a scientific, economic, and ethical perspective, it is crucial to employ animal models that accurately represent the endotype of the target patient population, not merely their clinical symptoms. These models must also account for intrinsic and extrinsic factors, like age, sex, metabolic status, and comorbidities, which impact OA's pathogenesis and its clinical and molecular variability and can profoundly influence not only structural and symptomatic disease severity and progression but also the underlying molecular pathophysiology. The molecular definition of the OA subpopulation must also be reflected in the read-outs, as the traditional methods—macroscopic and histological scoring, along with limited gene expression profiling of established biomarkers for cartilage degradation, extracellular matrix (ECM) turnover, and synovial inflammation—are inadequate for discovering new, phenotype- and endotype-specific biomarkers or therapeutic targets. Thus, animal model characterisation should evolve to include both clinically and pathophysiologically pertinent measures of disease progression and response to treatment. This review evaluates the utility and accuracy of current animal models in OA research, focusing on their capacity to replicate the disease’s pathophysiological processes.

**Graphical Abstract:**

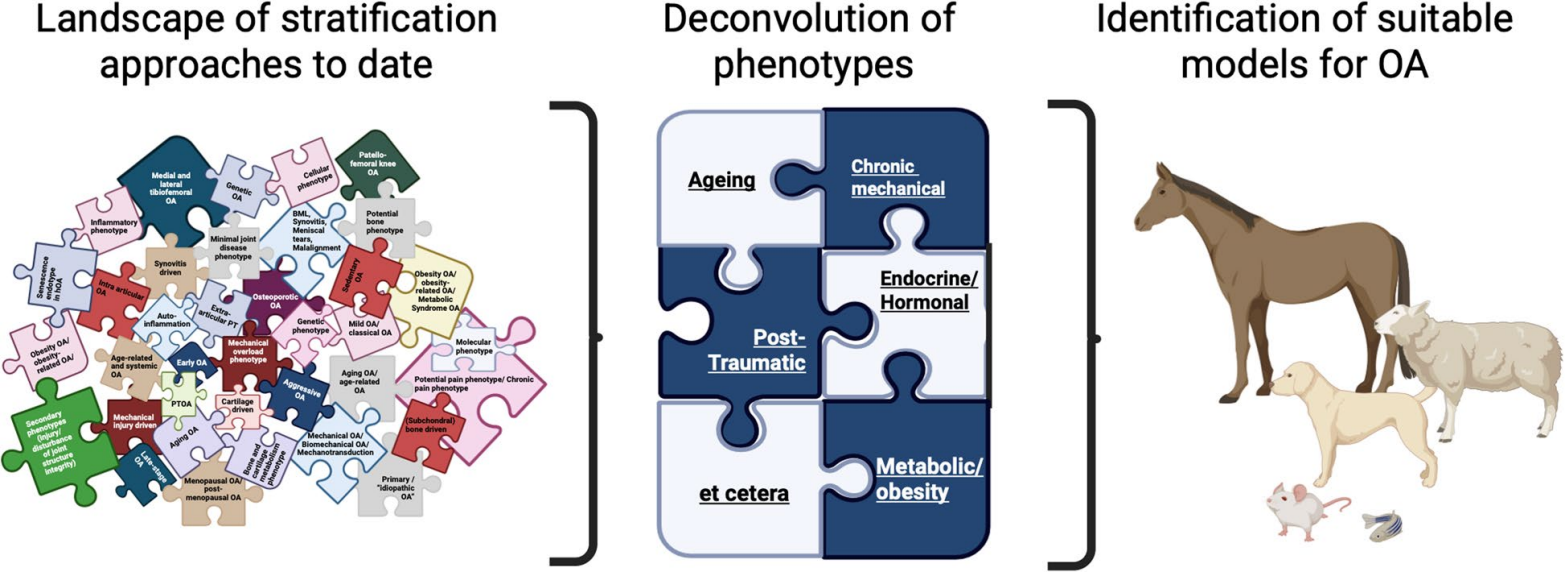

## Introduction

Osteoarthritis (OA) currently affects approximately 500 million people worldwide and is projected to become the leading cause of disability by 2030 [1–3]. Despite this widespread prevalence, there are still no curative treatments available, amplifying the burden on individuals, societies and healthcare systems alike. The notable absence of therapeutic breakthroughs can be attributed to several factors, with the lacking consideration of disease heterogeneity in preclinical and clinical trials prime among them [4–7].

OA is now recognized as a heterogenous syndrome comprising distinct subtypes, each arising from different aetiologies and involving diverse cellular, molecular, and biomechanical pathways. Despite this variability, these pathways ultimately converge in a common end-stage pathology characterized by similar clinical and radiographic features. Acknowledging this complexity paves the way for targeted therapies, which may overcome the current therapeutic limitations. However, while traditional treatments, limited to pain management at the symptomatic stage of the disease, were universally applicable to OA, the development of disease-modifying therapies requires the stratification of patients based not only on observable characteristics (phenotypes) but also on molecular pathomechanisms (endotypes) that may warrant distinct targeted therapeutic strategies [8]. Indeed, meta analyses revealed that only two of 199 reported candidate genes were consistently associated with OA, emphasizing the importance of appropriate categorization and selection of endotype-specific patient populations for successful biomarker and therapeutic target discovery [9–12]. Disease stratification and endotype-based personalized therapies have been successfully implemented in allergy and asthma treatment and are expected to also significantly enhance OA therapeutic success. Accordingly, over the past decade, there has been a surge in efforts to stratify OA. This has resulted in a multitude of proposed OA subtypes, variably defined by clinical and/or molecular characteristics [13, 14]. However, significant overlap exists between these subtypes, and further research is necessary to establish a unified framework of well-characterised molecular endotypes that correlate with distinct clinical phenotypes and therapies [13].

Diagnosing OA in its early stages remains challenging due to the insensitivity of current clinical diagnostic methods to pre-symptomatic cellular and molecular changes and the weak correlation between clinical symptoms and pathological changes [15–18]. Consequently, elucidating the molecular pathogenetic and pathophysiological events that occur during OA initiation and progression remains difficult, hindering the identification of therapeutic targets and biomarkers for early OA diagnosis and monitoring [19–21]. To address these limitations and account for the multifaceted disease mechanisms and the complex interaction between articular tissues and systemic influences, animal models are essential. However, a critical disconnect often exists between preclinical models and the OA patient population, hindering scientific progress and contributing to the low translational success rate of biomedical research. Notably, the prevalent use of young, normal-weight, male animals to model OA stands in massive contrast to the demographics of the human patient population, where ageing, obesity and female sex are predisposing factors [22]. Moreover, while age is the primary risk factor for OA and traumatic joint injury accounts for only 12% of cases [23], joint injury remains the most widely used method to induce OA in animal models. This discrepancy between experimental models and clinical reality is exacerbated by the distinct molecular pathophysiology of post-traumatic (secondary) OA compared to spontaneous OA [24].

Optimising the selection of animal models and the design of preclinical trials is essential to improving their predictive utility, minimising the number of animal lives that are avoidably wasted under the 3Rs principle [20, 22]. To facilitate the matching of OA endotypes with relevant in vivo models, this perspective review reflects on the utility and validity of currently available mammalian animal models in the context of their stratification into subtypes, focusing on ageing and senescence-driven, metabolic syndrome-associated and traumatic-injury-driven OA (see [13, 16, 25], for comprehensive discussions of other putative OA endotypes) [11, 14, 26, 27].

## General considerations for OA animal models

Evidence-based medicine, scientific rigour, and the 3Rs urgently require the careful selection of animal models based on the OA subtype of interest, the pathological feature of OA one wishes to investigate, and the study objectives. To improve the clinical translatability of preclinical findings, the chosen animal model should mimic the clinical and structural outcomes and molecular pathomechanisms of the OA phenotype and endotypes of interest, including the level of joint inflammation, cartilage and/or bone alterations as well as the severity and type of pain [19, 22, 24, 28–35]. This model-patient alignment also requires the consideration of intrinsic and extrinsic factors, such as age, metabolic status, sex, and co-morbid health conditions, that confound or contribute to OA pathogenesis and variability and can profoundly influence not only structural and symptomatic disease severity and progression but also the associated molecular pathophysiology [20, 36–57]. For example, the importance of age for the selection of animal models is demonstrated by studies investigating age-dependent responses to IL- 6 ablation, which is chondroprotective in young mouse models but can worsen age-associated OA in older mice [22]. Furthermore, even basal gene expression of a variety of joint tissues diverges between 12-week and 12-month-old mice, and medial meniscus destabilisation results in more severe OA in the older mice [22, 58]. The divergent outcomes in animal models representing different disease and patient characteristics highlight the importance of interpretation and translating preclinical trial results only within the framework of the specific OA subtype the model was selected to mimic.

In addition, an animal model should reflect the broad range and temporal progression of joint tissue pathology and allow quantifiable assessment of clinical outcomes. A fully sequenced and publicly available genome and proteome should be available to facilitate pathophysiological studies and the identification of treatment targets [19]. The species-specific variability in disease severity and time course following the same OA induction method is also a factor to be accounted for. Anterior cruciate ligament transection, as a frequently used OA induction method, induces slow progression to mild to moderate OA in sheep, goats and dogs but rapid progression to severe OA phenotype in mice [22, 59]. In addition, analogous to the distinct OA phenotype and endotypes observed in different appendicular joints in humans, different joints in animals show substantial variance in their response to the same insult [19, 60] which must be critically reflected during model selection.

Due to the heterogeneity of OA, there is no “gold-standard” animal model. Furthermore, as the pathophysiology of human OA is poorly understood and OA phenotype/endotype characterisation is still in progress, the validation of animal models against the human condition is difficult and typically limited to structural or biomechanical comparisons of articular cartilage [61], which fail to reflect the differences in pathogenetic mechanisms and corresponding OA phenotypes. Consequently, there are more than 50 different OA models utilising a wide range of species and disease induction approaches (see Table 1 for a detailed overview), each with a variety of advantages and shortcomings (Table 2). Commonly used species for OA research include foremost mice, which, together with guinea pigs, rabbits, and rats, represent over two-thirds of the animals used for OA research. Among large animals, dogs are most employed next to sheep, goats, and horses [19, 22, 29, 57]. OA in animal models can either arise spontaneously in naturally occurring and genetic models of disease or be induced using a variety of methods, including surgery, intra-articular chemical injection, mechanical overload, obesity, and high-fat diets [19]. Veterinary clinical populations suffering from naturally occurring disease are likely the best fit for a variety of phenotype and endotypes (post-traumatic, ageing, metabolic, etc.) of human OA, but are rarely used due to the time and resources required to recruit sufficient veterinary patients, inherently diverse patient populations, disease states and environmental conditions and the variable and protracted disease progression analogous to human disease. Veterinary patients with naturally occurring OA, following rigorous enrollment criteria and ethical protocols aligned with human clinical trials, could help bridge the gap between basic science and clinical application in humans, benefiting both animal and human patients.

**Table 1:** Overview of currently available in vivo models matched with the six specific OA phenotypes

Pathophenotype	Species	Short description
Ageing/senescence	Mouse [62–64] Guinea pig [65] Rabbit [66] Dog [67] Horse [68, 69] Pig [70] Cow [71]	Naturally occurring/spontaneous [62–72]
Inflammation/immune	- n.a. or unclear causalities for primary OA	- n.a. or unclear causalities for primary OA
Post-traumatic/acute impact event	Mouse [73–78] Rat [79–88] Guinea pig [89] Rabbit [90–92] Dog [75, 76] Sheep [93–99] Goat [100–102] Horse [103–107] Pig [108–113] Cow [114] Cat [115] Non-human primates [116]	Anterior cruciate ligament transection [89, 100] Partial/total medial meniscectomy [73, 87, 88, 93, 101, 116–119] Articular groove model/partial or full-thickness cartilage defect [75, 86, 105, 120] Intra-articular tibial plateau fracture [31, 77] Rupture via tibial compression overload [78] Medial meniscal destabilisation [81, 118] Medial meniscal tear [82–84, 92] Meniscal release [121] Partial and full-thickness osteochondral defect [104] Metacarpophalangeal ligament transection [122] or carpal fractures [123] Traumatic impact on the medial femoral condyle [124] Osteochondral defects and exercise [125] Partial meniscectomy and exercise [108] Cranial cruciate ligament transection [126]
Chronic mechanical overload/cumulative contact stress Lifestyle obesity Obesity-PT-OA progression	Mouse [40–42, 78] Rat [49, 87] Guinea pig [127, 128] Dog [129] Horse [130, 131] Pig [132] Non-human primates [133] Mouse [134, 135]	Cyclic articular cartilage tibial compression [40, 78] Monoiodoacetate [132]Collagenase [44, 136] Papain [46] Calcium pyrophosphatase crystals [137] Disuse/immobilisation of the Metacarpophalangeal joint followed by reuse and exercise [130, 131]
Endocrine/hormonal/metabolic syndrome	Mouse [138] Rat [139] Guinea pig [140] Rabbit [141] Sheep [142] Non-human primates [143]	Ovariectomy [138, 140–142]
Genetic	Mouse [74, 144] Zebrafish [145]	Collagen type 1 defect [144] Col2a1 deletion [146] Col9a1 knockout [147] Col10a1 knockout [145]

**Table 2:** Advantages and disadvantages of the currently used OA animal models

Species	Advantages	Disadvantages
Fish	• Easy breeding [145] • Short generation and maturation time [145] • Huge litter size [145] • Genetically defined [145] • Structural model of age-related OA [148] • Available genetic models [148, 149]	• Rel. expensive housing and special training [145] • Anatomical and biomechanical irrelevant (synovial joints) [145] • No naturally developing or surgically inducible OA model [145] • Small in size allows only limited sample volume collection [145]
Mouse and rat	• Inexpensive care and easy handling [32, 150] • Short breeding and maturation time-span [57, 151] • High informational potential due to fast disease progression [57] • Genetic predefinition [32] • A lot of available genetic models [150, 152] • A lot of available spontaneous [152] and inducible model [74] • Whole joint monitoring options [144, 153]	• Large anatomical- and physiological differences compared to humans [150] • Joint size-dependent operative and post-operative difficulties (decrease with increasing animal size) • [150] • Little to no potential for spontaneous degeneration of the knee joint [154]
Guinea pig	• Widespread models [155] • Low maintenance costs [155] • Fast maturation [150] • Natural disease development/sedentary lifestyle-dependent degeneration [156] • Similarity to human tissue pathology and biomarker concentration [150, 157]	• Great strain-dependent variability in disease progression [57] • Inexpedient model animal for joint overuse [150]
Rabbit	• Easy applicable [150] • Naturally [66] and inducible OA [90] • High similarity of human knee-joint anatomy [158] • Fully sequenced genome [159]	• Gain-deriving differences in biomechanical loading compared to human joints [158] • Slight structural alterations of joint tissues (vs. human joint) [57] • Difficult postoperative management [150]
Dog	• Strong resemblance to the macro- and microscopic level of the human joint anatomy [72] • Biochemical similarities of the intra-articular environment to human conditions [72] • OA derives naturally in multiple joints [119] • A lot of inducible models are available [121] • Useful species to identify novel biomarkers [160] • Human-similar treatment modalities [161] • Joint size allows for arthroscopic evaluations and synovial fluid withdrawal [162] • Good-fit model to study multiple phenotypes and endotypes [163],	• Strong emotional and ethical considerations [57] • High maintenance costs [57] • Divergent biomechanical properties from human conditions [72] • Joint-architectural alterations compared to human structures [72]
Sheep	• Manageable maintenance costs (vs. larger animals) [164] • Available inducible OA models [94–98, 142, 165] • Knee-joint anatomy and biomechanical properties are grossly similar to human conditions [164] • Best-fit model for translative studies involving the menisci [166] • Joint size allows for arthroscopic evaluations and synovial fluid as a screening tool [164] • Good-fit model to study multiple phenotypes and endotypes [164]	• OA appears very rarely [164] • Low sample size due to maintenance costs/animal [164] • Missing information about the sheep genome [164]
Horse	• Knee-joint anatomy and biomechanical properties are grossly similar to human conditions [167] • Joint size allows for arthroscopic evaluations [167] • Synovial fluid as a screening tool [167] • Good-fit model to study multiple phenotypes and endotypes [72] • Naturally and post-traumatic OA is a common clinical problem [68, 69] • High degree of structural and cellular commonality to the human osteochondral unit [150] • Sequenced genome available [168] • Inducible models are available [103, 104, 125]	• Strong emotional and ethical considerations [109] • Expensive housing and maintenance costs [59] • Specially trained personnel is required [59] • Low sample size due to high maintenance costs per animal [59] • The potential to develop OA naturally is strongly race-dependent [109]
Non-human primates	• Naturally occurring OA [169] • Inducible models are available [170] • Closest genetic inheritance to humans [169] • Joint size allows for arthroscopic evaluations and synovial fluid collection [169] • Available genome sequencing data [171]	• Enormous housing costs [169] • Difficulties in experimental management [169] • Structural differences in joint tissues [169] • Strong ethical considerations [169] • Genome availability varies between species [169]

OA represents a spectrum of molecularly distinct subtypes, but current classification efforts remain fragmented. While rheumatoid arthritis (RA) has benefited from endotype-specific classification based on transcriptomic and proteomic markers, OA endotyping is still in its infancy. A unified framework should integrate multi-omics data (genomics, transcriptomics, metabolomics, and proteomics) with imaging and clinical characteristics to establish reproducible OA endotypes. Studies have identified age-related differences in transcriptomic profiles of OA cartilage [172] and distinct metabolic signatures in synovial fluid of patients with different OA subtypes [173–175]. However, inconsistencies in patient cohorts and the lack of large-scale, standardized datasets hinder efforts to consolidate these findings. To address this, Artificial Intelligence (AI)-driven clustering methods could stratify OA into robust subtypes, analogous to approaches used in RA [16]. Establishing a global OA endotype database, akin to RA biomarker repositories, could facilitate cross-study validation and enhance preclinical model selection. Recent advances in omics technologies have revolutionized OA endotyping, enabling for instance precise molecular classification utilizing RNA sequencing and single-cell transcriptomics revealing distinct inflammatory, senescent, and metabolic endotypes [11]. Metabolomics and proteomics identify biochemical signatures in synovial fluid and serum [10]. Imaging tools like MRI and PET scans provide structural and metabolic insights, helping to correlate molecular changes with phenotypic severity [15]. Standardizing these methodologies will enhance cross-study comparability and preclinical-to-clinical translation. In addition to standard omics and imaging technologies, recent studies combine metabolomic profiling and machine learning algorithms to refine OA endotype classification further. For instance, Carlson et al. used synovial fluid metabolomics to identify metabolic phenotypes associated with structural cartilage changes, *suggesting distinct biochemical environments that underpin specific OA subtypes* [176]. Similarly, deep metabolic profiling of temporomandibular joint (TMJ) OA enabeled the identification of over 1,400 metabolites, including amino acids, lipids, and benzene derivatives, several of which correlated with disease severity [177]. Additionally, integrated metabolomic and transcriptomic analyses highlighted significant metabolic alterations, including disruptions in the tricarboxylic acid (TCA) cycle and amino acid metabolism in TMJ OA [178]. These findings underscore the potential of stage-related metabolic markers as non-invasive indicators of progression. Machine learning approaches, as shown by Nelson et al., who applied unsupervised clustering to biomarker data from the FNIH cohort, revealed progression-associated phenotypes that may inform patient stratification and therapeutic response [179]. Additionally, global metabolomic profiling has been shown to differentiate OA from other joint pathologies, offering diagnostic specificity at the biochemical level [180]. Spatial proteomic imaging (e.g., FTIR, Raman, nano-FTIR, MSI) supported mapping of cartilage and bone microenvironments at near-cellular resolution in a study conducted by Fan et al. [181]. These technologies captured biochemical gradients associated with inflammation, fibrosis, and mineralization. Combined with AI-based pattern recognition, these methods enable tissue-level phenotyping beyond histology. Rockel et al. introduced a variational autoencoder (OmicVAE) to integrate microRNA and metabolomic data from multiple biofluids [182]. This unsupervised model stratified patients into three molecularly defined endotypes, which predicted differential pain and function outcomes post-arthroplasty. Such integrative models demonstrate how latent omic features can outperform traditional clinical or single-omic classifiers in outcome prediction.

Similarly, Angelini et al. used clustering on biochemical markers from the IMI-APPROACH cohort to define three reproducible endotypes: *one with low tissue turnover (C1), a second driven by structural degradation (C2), and a thrid driven by systemic inflammation (C3)* [16]. These endotypes correlated with radiographic and symptomatic progression and were validated in an external cohort. The use of explainable AI (SHAP values) clarified key features per cluster, facilitating interpretability and potential trial stratification. The STEpUP OA consortium optimized a proteomic pipeline for synovial fluid (SF) using SomaScan aptamer technology [183]. An unprecedented number of SF samples (*n* = 1746) from diverse cohorts were analyzed, applying variance analyses (PCA, UMAP) revealing distinct proteomic patterns separating OA from injury. This provides a robust resource for future endotype discovery and supports SF as a local and disease-relevant matrix for biomarker identification. Mobasheri et al. emphasized the strategic importance of deep phenotyping using multi-omics and single-cell approaches to refine the cellular taxonomy of OA [1]. They advocate for harmonized data structures and open-access databases to integrate datasets from cartilage, bone, synovium, and immune cell compartments. Such efforts are key to redefining OA not as a single disease but as a set of overlapping molecular subtypes that may respond to targeted therapies.

These studies converge on a shared message, that endotype discovery in OA requires multimodal integration of omics data, spatial resolution, and computational modeling enabling fine-grained molecular phenotyping. They also allow reverse translation using human omics data to select or refine animal models that best reflect specific OA subtypes. They further shift the focus from morphological staging to mechanism-driven classification with the incentive to identify translatable biomarkers and therapeutic targets. A future OA classification framework should combine tissue-specific molecular signatures, joint-level imaging, and biofluid-derived biomarkers, supported by machine learning algorithms trained on harmonized datasets. Such a categorization may have the potential to improve patient stratification, refine animal models, and to accelerate disease-modifying drug development.

## Mind the patient: Do we have the right animal models for our human OA phenotypes and endotypes?

Overall, post-traumatic OA models are by far the most validated and accepted for their phenotype authenticity [152]. In contrast, the relevance and validity of models for other OA subtypes, for which the cause-and-effect relationship for disease onset and progression has not yet been unraveled, remain inherently ambiguous [184].

### Ageing and Senescence-driven OA

Ageing and cell senescence are associated with declining joint tissue functionality and regenerative capacity in cartilage and other tissues [22, 185, 186]. While ageing is the predominant risk factor for OA, the quiescent chondrocytes predominantly undergo stress-induced rather than replicative senescence due to accumulating damage from extrinsic and intrinsic stressors, such as reactive oxygen species (ROS) [187]. Accordingly, senescent cells have also been observed in articular tissues of young post-traumatic OA patients [188], highlighting the possible overlap of different endotypes that have to be considered in individualised targeted therapies.

Ageing and age-related diseases such as OA are characterised by cellular senescence and chronic systemic and local inflammation (inflammageing) and progenitor cell dysfunction [1]. Age-related increases in local and systemic proinflammatory mediators exacerbate cellular senescence of joint tissue-resident cells, such as chondrocytes and synoviocytes, and the subsequent release of senescence-associated secretory factors (SASP) with elevated pro-inflammatory cytokines [9–11, 22, 36, 185, 186, 189]. This, in turn, intensifies synovial inflammation, creating a vicious cycle where senescent cells promote inflammageing and inflammageing accelerates cellular senescence.

To accurately mimic ageing and senescence-driven OA, animal models should replicate the hallmarks of ageing, including senescence, inflammageing and progenitor cell exhaustion. Aging remains the predominant risk factor for OA, yet the pathophysiological mechanisms of aging-associated OA are underrepresented in preclinical models. However, in ageing research, models merely phenocopying selected ageing manifestations are often erroneously used to conclude the mechanisms of ageing [190]. Notably, a significant portion of studies cited for cellular senescence (63%) and stem cell exhaustion (62%) utilise models with unclear relevance to ageing [190]. Caution is warranted when using progeroid mouse models to study age-driven OA pathomechanisms. For example, Xpd^TTD^ mice, a commonly used progeroid model exhibiting severe osteoporosis, do not display accelerated cartilage ageing, underscoring the highly compartmentalised nature of ageing phenotypes in progeroid syndromes [191]. The STR/ORT (Strain 1/Old Retirement) mouse is one of the most widely studied spontaneous models for age-related OA, developing progressive joint degeneration without surgical induction, closely mimicking the multifactorial onset observed in humans [62]. The STR/ORT mouse model spontaneously develops OA. Disease onset typically begins between 18 and 24 weeks of age and presents with hallmark features such as cartilage erosion, osteophyte formation, and subchondral bone sclerosis [192, 193]. Importantly, STR/ORT mice display a polygenic susceptibility to OA, mimicking the multifactorial nature of human disease, and show upregulation of matrix metalloproteinases (e.g., MMP-3, MMP-13), chondrocyte hypertrophy, and reduced aggrecan content [40, 194]. Despite these strengths, the model has limitations. The murine cartilage matrix composition differs from humans in terms of glycosaminoglycan content and thickness, and mice experience distinct mechanical joint loading patterns due to quadrupedal gait and reduced body mass [57, 195]. Moreover, STR/ORT mice exhibit a marked sexual dimorphism, with males showing a higher and more consistent OA incidence most likely linked to androgenic signaling and growth plate dynamics [40, 196]. Although immune mechanisms are not the primary focus of this model, age-associated changes in immune cell profiles, including altered macrophage polarization and T cell senescence, have been reported in aged mice generally and may be relevant for STR/ORT pathogenesis [197, 198]. Overall, the STR/ORT mouse remains one of the few models that recapitulates slow, spontaneous OA progression in the absence of overt trauma, making it uniquely suited for studies on age-related joint degeneration. For instance, elderly STR/ORT mice were already used to explore the potential therapeutic potential of peptides targeting cartilage degradation. Improved cartilage integrity and decreasing expression of OA markers, MMP-13 and COL10A1, suggest that Gly-Arg-Gly-Asp-Ser (GRGDS) administration may enable ageing-related cartilage damage [199].

In contrast to genetically predisposed murine models such as the STR/ORT mouse, canine and equine models, which naturally develop OA due to age and mechanical stress, offer advantages in biomechanical similarity and joint size, allowing for longitudinal imaging and biomarker assessments akin to human patients [68]. Aged canines, particularly in larger breeds such as Labrador Retrievers or Beagles, frequently develop OA in the stifle and hip joints with age. Disease onset is often exacerbated by congenital dysplasia or joint instability but progresses in the absence of experimental induction. Histopathological features mirror human OA, including fibrillation and erosion of the articular cartilage, subchondral bone remodeling, and low-grade synovial inflammation. Notably, dogs exhibit gait patterns, joint kinetics, and cartilage thickness that are more similar to humans than rodents, enhancing their relevance for longitudinal imaging and therapeutic testing [200, 201]. In addition, aging dogs with OA diagnosis and PTOA models display exhibited levels of MMP- 13, IL- 1β, IL- 6, and TNF-α in synovial fluid and tissue, aligning with known catabolic cascades in human disease [202, 203]. Horses develop naturally occurring OA in high-load joints such as the carpus, fetlock, and tarsus, particularly in older performance horses. These animals present subchondral sclerosis, osteophyte formation, and early cartilage matrix changes, including depletion of proteoglycans and increased collagen II fragmentation [68]. Equine cartilage shares key features with human cartilage, such as comparable chondrocyte density, ECM structure, and cartilage thickness (~ 1.5–2 mm). Moreover, aged horses with OA show significant elevations of biomarkers such as CTX-II, COMP, and CPII, which are widely used in clinical trials for monitoring cartilage turnover [167, 204]. Their large joint size and tolerance for serial arthroscopy further enable longitudinal assessments, making them ideal for evaluating disease-modifying OA drugs (DMOADs) in aging contexts. Thus, animal models that spontaneously develop age-related joint tissue changes and naturally occurring OA offer distinct advantages for studying the complex interplay between senescence, inflammation, and joint degeneration, investigating the molecular pathomechanisms of the disease and identifying potential therapeutic targets [62, 63, 66–68]. However, challenges such as high cost, genetic variability, and ethical constraints limit their widespread use. Future research should focus on cross-validating spontaneous and induced models with human endotypes to refine model selection.

### Metabolic syndrome associated OA

Obesity doubles the risk of symptomatic OA in both weight-bearing (i.e., knee) and non-weight-bearing (i.e., hand) joints, indicating contributions beyond biomechanical overload with adipokine levels correlating with OA severity. Obesity triggers inflammation and remodeling of white adipose tissue, adipocyte hypertrophy and hyperplasia and the pro-inflammatory phenotype of adipose tissue-resident immune cells [205–207]. Altered adipokine signatures in obesity, especially with concurrent metabolic syndrome, are characterised by decreased levels of anti-inflammatory adipokines (e.g., adiponectin and omentin-1) and the upregulation of pro-inflammatory adipokines (e.g., leptin, resistin, and visfatin) and foster a pro-inflammatory milieu akin to inflammageing, driving metabolic syndrome-related chronic diseases, such as OA. In addition, metabolic syndrome is associated with an increased chronic cellular senescence burden, including senescence of mesenchymal stromal cells (MSCs), derived not only from fat but also from bone marrow [51, 52, 205–208].

Metabolically induced OA models are crucial for exploring the relationship between metabolism and OA development [56]. Beyond systemic inflammation, obesity-induced OA also involves intrinsic metabolic changes within articular cartilage, promoting disturbance in the intracellular lipid homeostasis [209]. A recent study performed by Liu et al. demonstrated that adipokines such as leptin and resistin not only exacerbate synovial inflammation but also directly alter chondrocyte lipid metabolism, leading to increased lipid droplet accumulation and oxidative stress [210]. This dysregulated fat metabolism impairs chondrocyte function, promoting catabolic enzyme activity (e.g., MMP-13) and accelerating cartilage breakdown. The high-fat diet-induced OA model effectively replicates these intra-articular changes, supporting its validity for studying metabolic OA endotypes [211, 212].

In addition to veterinary patients suffering from naturally occurring metabolic syndrome associated with OA, high-fat diet-induced OA has been established as a good model for metabolic OA [211]. It mimics not only the relevant OA pathogenesis characterised by local and systemic inflammation with elevation of specific cytokines, chemokines and adipokines but also induces typical general alterations, including anxiety and hyperalgesia, as well as decreased muscle function and locomotor activity [211, 213, 214]. These striking similarities to the human disease progression render this in vivo model for this particular OA subtype a valuable tool for basic and applied research [22, 184, 211, 213–217]. Nonetheless, a clear separation and stratification between metabolic and chronic mechanical overloading subtypes is difficult as global analysis of structure (number of lesions & Mankin score) and secretion profiles (i.e., adipokines) for obesity-related disease onset and progression in combination with mechanical altercations is heterogenous among study reports [184].

### Traumatic-injury driven OA

Acute trauma to joint structures, such as cartilage, subchondral bone, ligaments and meniscus, initiates molecular cascades leading to post-traumatic OA, exacerbating inflammatory and catabolic responses (e.g., ROS, toll-like receptor activation).

Post-traumatic OA in animal models can be induced through various methods, including non-invasive mechanical loading, surgical induction of cartilage lesions or surgical destabilisation of the joint, e.g. in the knee by transection of the cranial cruciate ligament, collateral ligaments, or meniscotibial ligament with or without removing all or part of the meniscus [79, 103, 108, 124, 218, 219]. Trauma-induced OA models mimic injuries also commonly observed in human patients and reflect the mechanical and biological changes observed in human OA, providing a controlled environment to study disease progression and possible therapeutic options. While surgical models offer the benefit of precisely targeting the tissue of interest, they may not fully capture the damage to other joint structures often seen in real-world injuries. For example, impact-induced bone bruises, evident in 80% of human ACL tears, are not typically replicated in surgical models [220, 221]. In addition, opening the joint introduces compounding effects by contributing to articular inflammation and pain [220]. Closed-joint impact models, on the other hand, damage multiple articular structures, offering a more realistic representation of clinical injuries, but they are inherently more variable due to the less controlled nature of the injury [220].

## Matchmaking of OA models against specific human phenotypes requires deeper analysis

As the value of animal models also depends on the reproducibility and validity of the outcome measures [36, 184], an expansion of the assessment method repertoire (e.g., omics approaches, pain assessment) is urgently required to maximise the usefulness of preclinical research. Interestingly, while multi-modal analyses and omics approaches have been successfully applied for molecular research in rheumatoid arthritis for over a decade, they are new to the OA research field. Currently, read-outs are typically limited to macroscopic and histological scoring as well as gene expression and protein secretion screening of a few established biomarkers of cartilage degeneration, ECM turnover, and synovial inflammation, such as collagen type 2 (Col2), type 1 (Col1), type 10 (Col10), matrix metalloproteinases (MMP)-1, -3 and -6, interleukin (IL)-1 and -6, and tumor necrosis factor-alpha (TNFa). The limitation of analysis approaches to well-established procedures and molecular indicators hampers the discovery of novel, phenotype, and endotype-specific biomarkers and therapeutic target identification. OA molecular pheno- and endotyping and model-OA-subtype matching can benefit from the increasing availability and affordability of omics methods and annotation of animal species [174], as shown by recent studies identifying age-related differences in murine post-traumatic knee OA transcriptome and metabolomic characteristics of anterior cruciate reconstructed versus sham-operated ovine synovial fluid [172, 174]. In general, a comprehensive molecular characterisation of phenotypes in animals and humans will help to identify where great overlaps exist, which in turn will indicate the most suitable animal models for preclinical studies. Once the appropriate animal model is identified, the next step is to successfully treat the condition in this model. Following the success in preclinical studies, the corresponding patient cohort can be targeted for clinical trials.

Additionally, it may be beneficial to revisit previously unsuccessful clinical trials. By redefining patient sub-cohorts, there is an opportunity to reanalyse the data to determine if the treatment might have significant effects that were previously overlooked. This iterative approach can refine the understanding and improve the success rates of clinical interventions.

As pain relief and joint function are the most important outcomes for clinicians and patients, more emphasis should be placed on pain assessment in OA models. Regular pain assessment using species-specific pain scores (i.e., grimace scale), weight-bearing, functional gait analysis, and lameness scores should be mandatory for each preclinical trial to ensure proper analgesia for the animals and provide clinically relevant read-outs of OA progression. In addition, anxiety and depression are increasingly used to monitor OA symptoms and progression also in animals [222]. For example, in mice that underwent partial medial meniscectomy as a disease trigger to mimic human post-traumatic OA, a longitudinal, multiparametric assessment of pain revealed distinct time-dependent and disease-progression-related pain levels and mechanisms [73]. Another comprehensive study investigating the association between OA disease phenotypes, joint pathology, gene expression, and pain behaviour revealed phenotype-specific pain and peripheral sensory neuronal responses [163]. By combining gene expression analysis with a wide range of pain evaluation modalities, the study demonstrated that the molecular pathophysiology of pain and joint-tissue pathology is influenced by the underlying disease model even in the later stages of the disease. This highlights the importance of considering phenotype- and disease-stage-specific factors when interpreting animal model studies and extrapolating their findings to human disease. Additionally, the study emphasises the significance of pain assessment for animal welfare and relevant clinical outcomes [163].

In rheumatoid arthritis (RA) research, multi-modal analyses have significantly advanced our understanding of disease mechanisms, patient stratification, and treatment responses [5, 223, 224]. These approaches integrate various data types, such as genomic, transcriptomic, proteomic, and imaging data, to provide a comprehensive view of the disease. One prominent example is the integration of single-cell RNA sequencing (scRNA-seq) and single-cell chromatin accessibility profiling (scATAC-seq) to dissect the transcriptional and epigenetic landscape of synovial fibroblast subpopulations [225–228]. This combined strategy revealed conserved gene regulatory networks across human RA tissue and mouse models, highlighting distinct fibroblast endotypes that drive inflammation and joint damage [229]. In parallel, photoacoustic imaging combined with ultrasound (PA-US) has emerged as a powerful tool to non-invasively assess inflammatory activity in RA joints, capturing both vascular changes and tissue structure with high resolution [230–235]. Moreover, machine learning techniques, such as dynamic deep neural networks, have been employed to integrate clinical and biomarker data for more accurate prediction of RA progression and treatment response [236, 237]. These multi-modal approaches, linking molecular, cellular, imaging, and computational layers, have redefined disease stratification in RA and provide a conceptual and technical framework for OA endotyping.

Histopathological grading remains a cornerstone in OA research; however, traditional scoring systems like OARSI and Mankin exhibit critical limitations. These semi-quantitative methods are inherently subjective, relying on visual interpretation of morphological features such as cartilage surface integrity, matrix staining, and cellularity, which leads to considerable inter-observer variability and poor reproducibility [238–241]. Moreover, they predominantly assess late-stage structural changes in cartilage and fail to integrate molecular, cellular, or biomechanical alterations that are essential to distinguishing OA endotypes [25]. Artificial intelligence-driven histopathology and deep learning-based imaging analysis are efforts to refine histopathological assessment and to modernize histopathological evaluation in general [242].

Studies in RA have demonstrated AI’s ability to integrate histopathological, omics, and imaging data for more accurate disease classification [5, 239, 241]. Similarly, AI-powered deep learning models have achieved 93% accuracy in grading knee OA from radiographic images, significantly outperforming human raters [240]. Such methods could be applied to automate OA scoring systems, integrating cellular-level changes with structural histopathology for a more comprehensive disease assessment.

## Conclusion & Prospects

The poor reproducibility of biomedical research and lack of translatability of basic science to clinical applications call for a critical evaluation of preclinical models regarding their alignment with the clinical trial population’s OA phenotype and endotype, age, sex, confounding comorbidities, and evaluation parameters [22]. To successfully identify biomarkers and therapeutic targets for novel treatment approaches, animal models need to accurately mimic the aspects of human OA pathophysiology that are relevant to the OA subtype of interest and the study objectives. Longitudinal and multiparametric assessment of biomarkers in a variety of tissues, in serum, urine, or synovial fluid, will provide more meaningful information about the disease stage, structural pathology, and underlying molecular mechanisms. For example, the integration of AI-driven tools into OA classification and model evaluation offers a promising route to enhance the fidelity and translatability of preclinical research. Deep learning approaches applied to radiographic and histological images have demonstrated superior accuracy and consistency compared to human-assisted scoring, particularly in the grading of structural joint changes. However, their full potential lies beyond automation. To modernise histopathological OA scoring, a multi-dimensional refinement is warranted: a) the incorporation of molecular biomarkers into standard grading systems would enable a more mechanistic understanding of histological changes and better reflect the underlying disease biology; b) AI-based digital pathology pipelines could support automated, high-throughput, and reproducible analysis of tissue samples, including the identification of histological signatures specific to OA phenotypes; and c) machine learning models for multimodal data integration can combine histological imaging with transcriptomic, proteomic, and imaging datasets, supporting phenotype- and endotype-specific classification. By transitioning from morphology-based assessments to AI-enhanced, biomarker-informed classification systems, histopathology can become a more powerful tool for aligning animal models with specific human OA subtypes. This paradigm shift would not only improve model selection but also enhance the resolution of treatment effects, biomarker discovery, and translational success. The currently predominant evidence-based selection of animal models subtypes of interest is currently hindered by ambiguous definitions of the different OA patient subtypes and inadequate characterisation of existing animal models, which is typically limited to radiographic, macroscopic, and histological features and qPCRs of a few select ECM factors, proteases, and inflammatory mediators. However, our growing understanding of the cellular and molecular mechanisms of OA, combined with technical advancements in molecular imaging and omics technologies, is rapidly expanding the repertoire of methods available for the pathophysiologic stratification of both human patients and animals. This allows for a more accurate classification based on the underlying molecular mechanisms of the disease [20]. Hence, the characterisation of animal models should progress to include the criteria used to differentiate between different OA phenotypes and endotypes [243]. Similarly, clinically and pathophysiologically relevant readouts of disease progression and treatment response, and corresponding reporting guidelines, need to be established and standardised for models of each OA subtype. In addition to standardised evaluation criteria, consolidation of the current plethora of animal models may also aid in improving the comparability of preclinical data. Currently, post-traumatic knee OA, for example, is induced in species including mice, rats, rabbits, dogs, sheep, goats, and horses, using either non-invasive mechanical loading, surgical induction of cartilage lesions, or surgical destabilisation of the joint by transection of the cranial cruciate ligament, collateral ligaments, or meniscotibial ligament, with or without removing all or part of the meniscus. This profusion of models, all aiming to mimic the same OA subtype, spreads the data characterising each model thin and limits the comparability of study results. Limiting the number of models to those best reflecting each OA phenotype and endotype would greatly increase research synergies and hence contribute to reducing the number of animals needed for OA research, optimising research economy and ethics. Using reverse translation as a fidelity check will ensure that molecules and pathways identified in vitro and in vivo models of OA can reflect the naturally occurring pathology and could help identify the best-suited model species and OA induction methods. Uniform reporting standards of preclinical trials should also document and publish negative study outcomes. Initiatives such as the ‘One Health Initiative’ [89] can further facilitate the multi-directional flow of knowledge and synergistic gains for multiple disciplines, which will contribute to a better and more holistic interpretation and validation of data in the field of OA as well as OA phenotyping.

## Data Availability

No datasets were generated or analysed during the current study.

## Abbreviations

OA: Osteoarthritis
PTs: Phenotype
ETs: Endotype
CT: Computed tomography
MRI: Magnetic resonance imaging
Col2: Collagen type 2
MMP: Matrix metalloproteinase
IL: Interleukin
TNFa: Tumor necrosis factor-alpha
SASP: Senescence-associated secretory phenotype
RA: Rheumatoid arthritis
SF: Synovial fluid
TCA: The tricarboxylic acid cycle
TMJ: Temporomandibular joint
DMOADs: Disease-modifying osteoarthritis drug
FTIR: Fourier transform infrared spectroscopy
MSI: Mass spectrometry imaging
PCA: Principal Component Analysis
UMAP: Uniform Manifold Approximation and Projection
SHAP: Shapley Additive exPlanations
3Rs: Replacement, Reduction and Refinement
ACL: Anterior cruciate ligament
AI: Artificial intelligence
COMP: Cartilage Oligomeric Matrix Protein
ECM: Extracellular matrix
MSCs: Mesenchymal stromal cells
OARSI: Osteoarthritis Research Society International
PT: Post traumatic
ROS: Reactive oxygen species
qPCR: Quantitative polymerase chain reaction
